# Butyrate and Forskolin Augment Host Defense, Barrier Function, and Disease Resistance Without Eliciting Inflammation

**DOI:** 10.3389/fnut.2021.778424

**Published:** 2021-10-27

**Authors:** Kelsy Robinson, Qing Yang, Hong Li, Long Zhang, Bridget Aylward, Ryan J. Arsenault, Guolong Zhang

**Affiliations:** ^1^Department of Animal and Food Sciences, Oklahoma State University, Stillwater, OK, United States; ^2^Poultry Production and Product Safety Research Unit, United States Department of Agriculture (USDA)–Agricultural Research Service, Fayetteville, AR, United States; ^3^College of Animal Science and Technology, Henan Agriculture University, Zhengzhou, China; ^4^Institute of Ecology, China West Normal University, Nanchong, China; ^5^Department of Animal and Food Sciences, University of Delaware, Newark, DE, United States

**Keywords:** antimicrobial resistance, antibiotic alternatives, antimicrobial peptides, host defense peptides, necrotic enteritis

## Abstract

Host defense peptides (HDPs) are an integral part of the innate immune system with both antimicrobial and immunomodulatory activities. Induction of endogenous HDP synthesis is being actively explored as an antibiotic-alternative approach to disease control and prevention. Butyrate, a short-chain fatty acid, and forskolin, a phytochemical, have been shown separately to induce HDP gene expression in human cells. Here, we investigated the ability of butyrate and forskolin to induce the expressions of chicken HDP genes and the genes involved in barrier function such as mucin 2 and claudin 1 both *in vitro* and *in vivo*. We further evaluated their efficacy in protecting chickens from *Clostridium perfringens*-induced necrotic enteritis. Additionally, we profiled the transcriptome and global phosphorylation of chicken HD11 macrophage cells in response to butyrate and forskolin using RNA sequencing and a kinome peptide array, respectively. Our results showed a strong synergy between butyrate and forskolin in inducing the expressions of several, but not all, HDP genes. Importantly, dietary supplementation of butyrate and a forskolin-containing plant extract resulted in significant alleviation of intestinal lesions and the *C. perfringens* colonization in a synergistic manner in a chicken model of necrotic enteritis. RNA sequencing revealed a preferential increase in HDP and barrier function genes with no induction of proinflammatory cytokines in response to butyrate and forskolin. The antiinflammatory and barrier protective properties of butyrate and forskolin were further confirmed by the kinome peptide array. Moreover, we demonstrated an involvement of inducible cAMP early repressor (ICER)-mediated negative feedback in HDP induction by butyrate and forskolin. Overall, these results highlight a potential for developing butyrate and forskolin, two natural products, as novel antibiotic alternatives to enhance intestinal health and disease resistance in poultry and other animals.

## Introduction

Antimicrobial resistance is a major threat to human health (1). Host-directed therapy has emerged as a promising antibiotic-free strategy for disease control and prevention (2, 3). Host defense peptides (HDPs), also known as antimicrobial peptides, are small molecules of the innate immune system featuring antimicrobial and immunomodulatory properties (4, 5). Inducing HDP synthesis is a host-directed antimicrobial therapy that is being actively explored for human and livestock applications (2, 6, 7). HDPs are classified into two major families, namely defensins and cathelicidins, in vertebrate animals (4, 5). Defensins contain characteristic disulfide bridges and six cysteine residues that categorize them as α-, β-, or θ-defensins (8), while cathelicidins are identified by the presence of a conserved cathelin precursor (9). The chicken genome encodes a total of 14 β-defensins known as avian β-defensin 1–14 (*AvBD1*–*14*) and four cathelicidins known as *CATH1-3* and *CATHB1*, with their expression detected throughout the gastrointestinal (GI), respiratory, reproductive, and urogenital tracts as well as in the bone marrow and several types of immune cells (10, 11).

Butyrate is a short-chain fatty acid produced primarily by bacterial fermentation in the GI tract (12, 13). Butyrate induces HDPs mainly by acting as a histone deacetylase inhibitor (HDACi) to enhance acetylation of histones, relaxation of gene promoters, and transcription of target genes (14). Forskolin (FSK) is a natural product belonging to the diterpene family extracted from the roots of *Coleus forskohlii*, a member of the mint family grown in India, Nepal, and Thailand that is traditionally used to treat various inflammation-related disorders (15). FSK acts as a natural adenylyl cyclase agonist to increase the synthesis of cyclic adenosine monophosphate (cAMP) (15), which in turn activates protein kinase A (PKA) resulting in phosphorylation and activation of a transcription factor known as cAMP responsive element (CRE)-binding protein (CREB) (16). Activated CREB then binds to a CRE region of its target gene promoter leading to increased gene transcription. FSK-mediated HDP gene induction is believed to transduce through the cAMP-PKA signaling pathway (17, 18). It is well-known that a negative feedback loop exists for the cAMP-PKA pathway through upregulation of inducible cAMP early repressors (ICER) that represent a group of smaller proteins transcribed alternatively from the CRE modulator (*CREM*) gene (19). ICER suppresses gene transcription targeted by the cAMP-PKA-CREB pathway by competing with CREB for the CRE region of target gene promoters (19).

Integrity of mucosal barrier function is critical for the host to achieve homeostasis and defend against external insults from the environment (20). Barrier function is maintained mainly by the mucus layer and tight junctions (20). Mucin-2 (MUC2) constitutes a major component of the mucus in the GI tract (21), while tight junctions are formed by a network of transmembrane and cytosolic adaptor proteins such as claudin-1 (CLDN1) and zonula occludens-1 (ZO1) that join together the cytoskeletons of neighboring cells (22). Butyrate is capable of enhancing barrier function (12, 13), but the role of FSK in barrier function remains unknown.

We previously found butyrate and FSK to be capable of inducing chicken *AvBD9* gene expression in a synergistic manner (18). In this study, we continued to investigate the role of butyrate and FSK in the expressions of other chicken HDPs both *in vitro* and *in vivo*. We also examined their impact on barrier function. Necrotic enteritis (NE), caused by *Clostridium perfringens*, is an economically devastating enteric disease and often associated with growth retardation, reduced feed efficiency, small intestinal pathology, and 10–20% mortality in chickens (23). The efficacy of dietary supplementation of butyrate and FSK in protecting chickens from NE was evaluated. The mechanism of action of the HDP-inducing synergy between butyrate and FSK was further explored using RNA sequencing and a chicken-specific kinome peptide array. We revealed that butyrate and FSK synergistically enhanced innate host defense, barrier function, and disease resistance without triggering inflammation and that ICER is a major negative regulator of butyrate- and FSK-induced HDP gene expression.

## Materials and Methods

### Cell Culture and Stimulation

Chicken HD11 macrophage cells (24) were maintained in Roswell Park Memorial Institute (RPMI) 1640 (Hyclone, Logan, UT) supplemented with 1% penicillin/streptomycin and 10% fetal bovine serum (Atlanta Biologicals, Lawrenceville, GA). After overnight seeding in 12-well plates at 1 × 10^6^ cells/well, HD11 cells were stimulated with different concentrations of sodium butyrate (MilliporeSigma, St. Louis, MO) and FSK (Santa Cruz Biotechnology, Santa Cruz, CA) individually or in combination. Cells were subjected to total RNA isolation for subsequent quantitative reverse transcription PCR (RT-qPCR) or RNA sequencing as described below.

### Dietary Supplementation of Butyrate and FSK to Chickens

A total of 60 day-old male broiler chickens were obtained from Cobb-Vantress Hatchery (Siloam Springs, AR), divided randomly into six groups of 10, and provided *ad libitum* with tap water and a non-medicated commercial basal diet (DuMOR Chick Starter/Grower 20%, SKU #507821099, Tractor Supply Co., Brentwood, TN). Animals were raised in santitized floor pens with fresh pine wood shavings under standard management for 4 days. While one group of animals were allowed to continue on the basal diet, five other groups were switched to experimental diets supplemented with 1 g/kg microencapsulated sodium butyrate (CM3000® containing 30% pure sodium butyrate, King Techina, Hangzhou, China), 20 mg/kg *C. forskohlii* (CF) extract containing 10% FSK (Vitacost, Lexington, NC), or a combination of 1 g/kg CM3000® and CF extract (5, 10, or 20 mg/kg feed). After 2 days of feeding, eight chickens from each group were euthanized for collection of the jejunum at −80°C until homogenization for RNA extraction. All animal procedures were approved by the Institutional Animal Care and Use Committee of Oklahoma State University under protocol AG1610.

### RT-qPCR Analysis of Gene Expression

Total RNA was isolated from chicken HD11 cells or the jejunal segments using RNAzol RT (Molecular Research Center, Cincinnati, OH) according to the manufacturer's protocol. RNA was quantified using Nanodrop 1000 (Nanodrop Products, Wilmington, DE) and the ratios of A_260_/A_230_ and A_260_/A_280_ were used to indicate RNA quality. Reverse transcription was performed using Maxima First Strand cDNA Synthesis Kit (ThermoFisher Scientific, Pittsburgh, PA), followed by qPCR analysis with QuantiTect SYBR Green PCR Kit (Qiagen, Valencia, CA) in 10-μL reactions. The qPCR protocol consisted of an initial activation at 95°C for 10 min and 40 cycles of 94°C for 15 s, 55°C for 20 s, and 72°C for 30 s. Melting curve analysis was performed to determine PCR specificity, and relative fold changes were estimated with the ΔΔCt method (25) using glyceraldehyde 3-phosphate dehydrogenase (*GAPDH*) as the reference gene for data normalization as we previously described (18, 26).

### Chicken Model of Necrotic Enteritis

A total of 48 male day-of-hatch Cobb broiler chicks were obtained from Cobb-Vantress Hatchery and randomly assigned to four floor pens with 12 animals per pen. Birds were raised for 10 days on a non-medicated basal diet (DuMOR Chick Starter/Grower 20%, Tractor Supply Co.) under standard management. On day 10, chicks were weighed individually and 36 of similar body weights were transferred to battery cages with three cages per treatment and three animals per cage. Upon transfer, one group of animals continued with the basal DuMOR diet, while the other three groups were supplemented with 1 g/kg microencapsulated sodium butyrate (CM3000®, King Techina), 5 mg/kg CF extract (Vitacost), or a combination of 1 g/kg CM3000® and 5 mg/kg CF extract. Following a 3-day feeding and acclimatization, chickens were fasted overnight prior to daily challenges with the *netB*- and *tpeL*-positive *C. perfringens* strain Brenda B (provided kindly by Dr. Lisa Bielke at the Ohio State University) (27) to induce NE as described (28). *C. perfringens* was cultured sequentially in cooked meat medium (ThermoFisher Scientific) and fluid thioglycollate (ThermoFisher Scientific) broth every 18 h. Bacterial challenge was performed by feeding a 1:1 mixture of bacterial overnight culture and respective diets twice daily for 4 days. On day 19, all animals were euthanized by CO_2_ asphyxiation and necropsied to determine the duodenal and jejunal lesion scores in a blind manner according to a 6-point scoring system as described (29). A segment of the jejunum was also fixed in formalin (VWR International, Radnor, PA), processed, and stained with hematoxylin and eosin (VWR International). Additionally, the jejunal digesta was collected and the *C. perfringens* titer was determined using qPCR as described below.

### Quantification of *C. perfringens*

Microbial DNA was isolated from the jejunal digesta using the ZR Fecal DNA Miniprep Kit (Zymo Research, Irvine, CA). DNA quality and quantity were determined using Nanodrop 1000. The *C. perfringens* titer was determined using a standard curve-based qPCR and *C. perfringens*-specific primers as described (30, 31). The standard curve was constructed using 10-fold serial dilutions of genomic DNA isolated from a known count of pure *C. perfringens* culture. The qPCR protocol consisted of an initial activation at 95°C for 10 min, followed by 40 cycles of 94°C for 15 s, 60°C for 20 s, and 72°C for 30 s. Melting curve analysis was performed to determine PCR specificity and the *C. perfringens* titer was calculated as colony-forming units (CFU)/g of the jejunal digesta.

### Western Blot Analysis

Chicken HD11 cells were treated in duplicate with butyrate (2 mM) or FSK (5 or 200 μM) individually or in two different combinations for 6 h. Cells were then lysed in 0.5 mL of TNT buffer (20 mm Tris, pH 7.5, 200 mm NaCl, 1% Triton X-100) containing a cocktail of protease inhibitors (MilliporeSigma). Proteins (50 μg) in the supernatants were separated on 12% SDS-PAGE, transferred to an Immobilon-P® polyvinylidene difluoride membrane (MilliporeSigma), and blotted sequentially with a rabbit anti-CREM/ICER polyclonal antibody (MilliporeSigma, #AV34777; diluted 1:500) and a goat anti-rabbit IgG secondary antibody conjugated with horseradish peroxidase (MilliporeSigma; diluted 1:2,000). The reactive bands were visualized using enhanced chemiluminescence (ThermoFisher Scientific).

### RNA Sequencing and Analysis

Chicken HD11 cells were treated in duplicate with or without 2 mM butyrate, 5 μM FSK, or in combination for 24 h. Total RNA was isolated for cDNA library preparation using TruSeq RNA Sample Prep Kit (Illumina, San Diego, CA), followed by RNA sequencing by Novogene (Beijing, China) on an Illumina HiSeq 2000 platform. The raw sequence reads were filtered using Cutadapt (32) and Prinseq (33). High-quality clean reads from each sample were separately aligned to the chicken genome assembly (Ensembl, Gallus gallus 5.0) using TopHat2 (34). The resulting transcript abundances were quantified using a software package, RNA-Seq by Expectation Maximization (RSEM) (35). The expression levels were determined based on the values of fragments per kilobases per million mapped reads (FPKM). Differentially expressed genes with >2-fold difference (relative to the control) and false discovery rate (FDR) <0.05 were identified using edgeR (36) and visualized on a heat map using MultiExperiment Viewer (MeV) (37). To identify enriched gene ontology (GO) terms and Kyoto Encyclopedia of Genes and Genomes (KEGG) pathways, functional enrichment of the genes was analyzed using a web-based tool, KEGG Orthology-Based Annotation System, intelligent version (KOBAS-i) (38). Only the GO terms and KEGG pathways with *P* < 0.05 were considered significantly enriched. Venn diagram was generated using Venny 2.0 online software (https://bioinfogp.cnb.csic.es/tools/venny/index.html).

### Kinome Peptide Array and Analysis

Chicken HD11 cells were treated in duplicate with or without 2 mM butyrate, 5 μM FSK, or in combination for 4 h. Cell pellets were lysed using 100 μL lysis buffer (20 mM Tris–HCl pH 7.5, 150 mM NaCl, 1 mM ethylenediaminetetraacetic acid (EDTA), 1 mM ethylene glycol tetraacetic acid (EGTA), 1% Triton X-100, 2.5 mM sodium pyrophosphate, 1 mM Na_3_VO_4_, 1 mM NaF, 1 μg/mL leupeptin, 1 g/mL aprotinin, and 1 mM phenylmethylsulphonyl fluoride; all purchased from MilliporeSigma, St. Louis, MO). The supernatants were subsequently subjected to a chicken-specific kinome peptide array procedure as described (39) with slight modifications (40). The kinome peptide array was constructed to contain a total of 771 chicken-specific peptides that were derived from phosphorylation sites of 572 proteins. Data normalization and clustering analysis were performed as described (41). GO terms and KEGG pathway analysis were performed by uploading a list of statistically significant peptides (*P* < 0.001) to the Search Tool for the Retrieval of Interacting Genes (STRING) (42).

### Statistical Analysis

For cell culture experiments, the results were shown as means ± standard error of the mean (SEM) of 3–4 independent experiments. Following the confirmation of normality of the data with Shapiro-Wilk test, statistical significance was determined using one-way ANOVA with *post-hoc* Tukey's test in Prism (GraphPad Software, La Jolla, CA). Statistical significance was considered if *P* < 0.05. Treatments with *P* < 0.20 were also indicated to show a trend.

## Results

### Butyrate and FSK Synergistically Induce Chicken HDP and Barrier Function Genes

**Figure 1:**
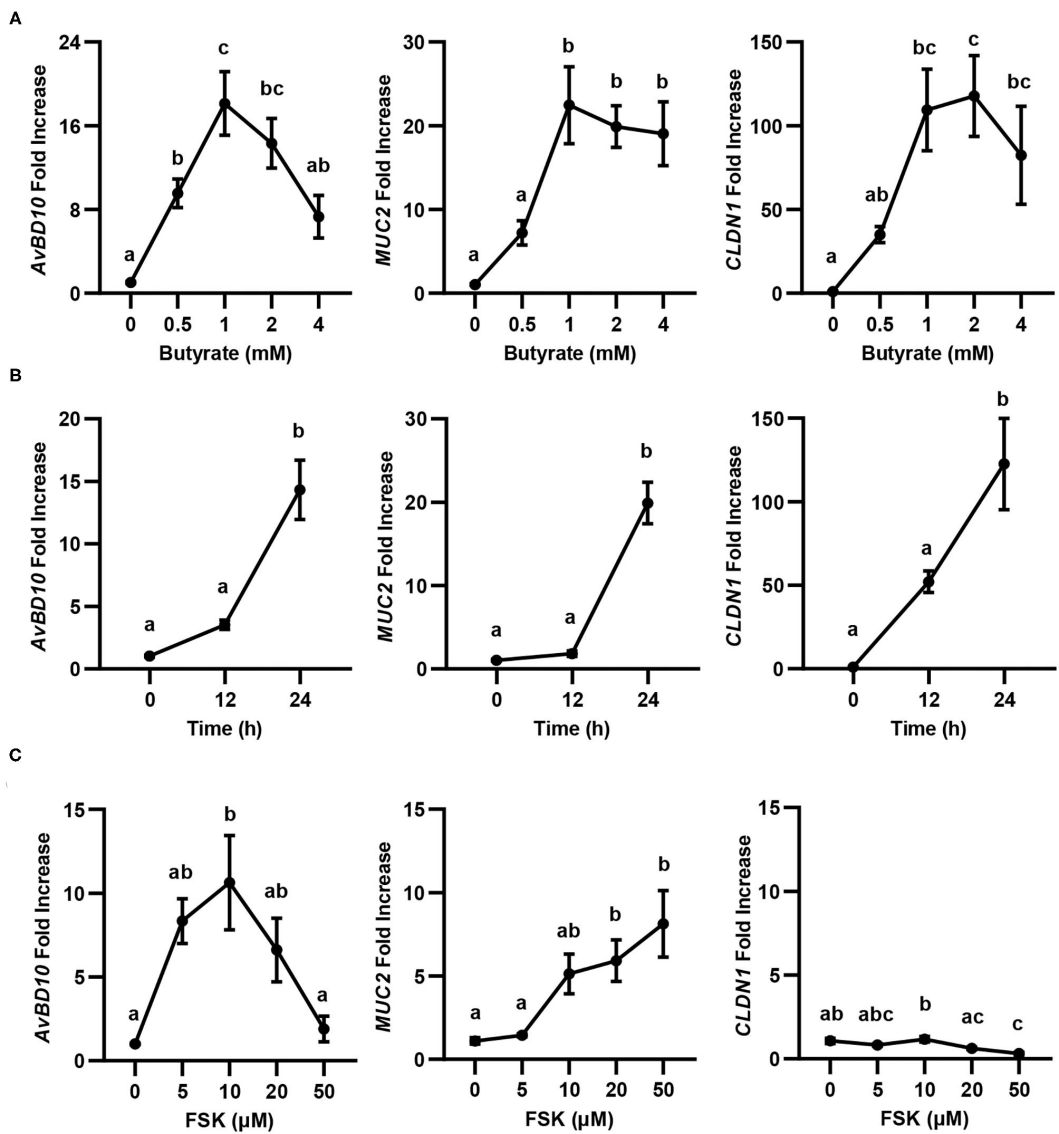
Concentration- and time-dependent induction of avian β-defensin 10 (*AvBD10*), mucin 2 (*MUC2*), and claudin 1 (*CLDN1*) in macrophages by butyrate and forskolin (FSK). Chicken HD11 cells were stimulated in duplicate with different concentrations of sodium butyrate or FSK for 24 h, or 2 mM butyrate for 12 or 24 h, followed by RT-qPCR analysis of gene expression. **(A)** Concentration-dependent gene induction in response to butyrate for 24 h. **(B)** Time-dependent gene induction in response to 2 mM butyrate. **(C)** Concentration-dependent gene induction in response to FSK for 24 h. Results are shown as means ± SEM of 3–4 independent experiments. Statistical significance (*P* < 0.05), denoted by non-common letters, was determined using one-way ANOVA and *post-hoc* Tukey's test.

Butyrate is known to synergize with FSK in inducing chicken *AvBD9* gene expression (18). To study whether the synergy also occurs with other HDP genes as well as two major genes involved in barrier function (*MUC2* and *CLDN1*) (20), we first conducted both dose-response and time-course experiments in chicken HD11 macrophage cells with butyrate and FSK separately, followed by RT-qPCR analysis of gene expression. Similar to *AvBD9* (18), *AvBD10, MUC2*, and *CLDN1* were all induced by butyrate in both concentration- (Figure 1A) and time-dependent manners (Figure 1B). A peak induction of all three genes was observed with 1 mM butyrate at 24 h. A longer duration was not attempted because of much reduced cell numbers and hence RNA concentrations. Besides *CLDN1*, two other tight junction protein genes tested (*CLDN5* and *ZO1*) were also significantly induced by butyrate (data not shown). Notably, higher concentrations of butyrate led to diminished inductions of all three genes examined (Figure 1A), presumably due to the presence of a negative feedback mechanism. FSK also showed a dose-dependent induction of *AvBD10* and *MUC2*, but not *CLDN1* (Figure 1C). Similar to *AvBD9* (18), a maximum induction of *AvBD10* by FSK occurred at 10 μM and higher concentrations showed a reduced response, while 50 μM FSK gave the strongest upregulation of *MUC2* gene expression (Figure 1C). *CLND1* failed to be induced by FSK at any concentrations tested, and was in fact significantly suppressed by 50 μM FSK (Figure 1C). Time-course experiments with FSK were not attempted because 24 h was known to be optimal in giving a peak induction of *AvBD9* (18).

**Figure 2:**
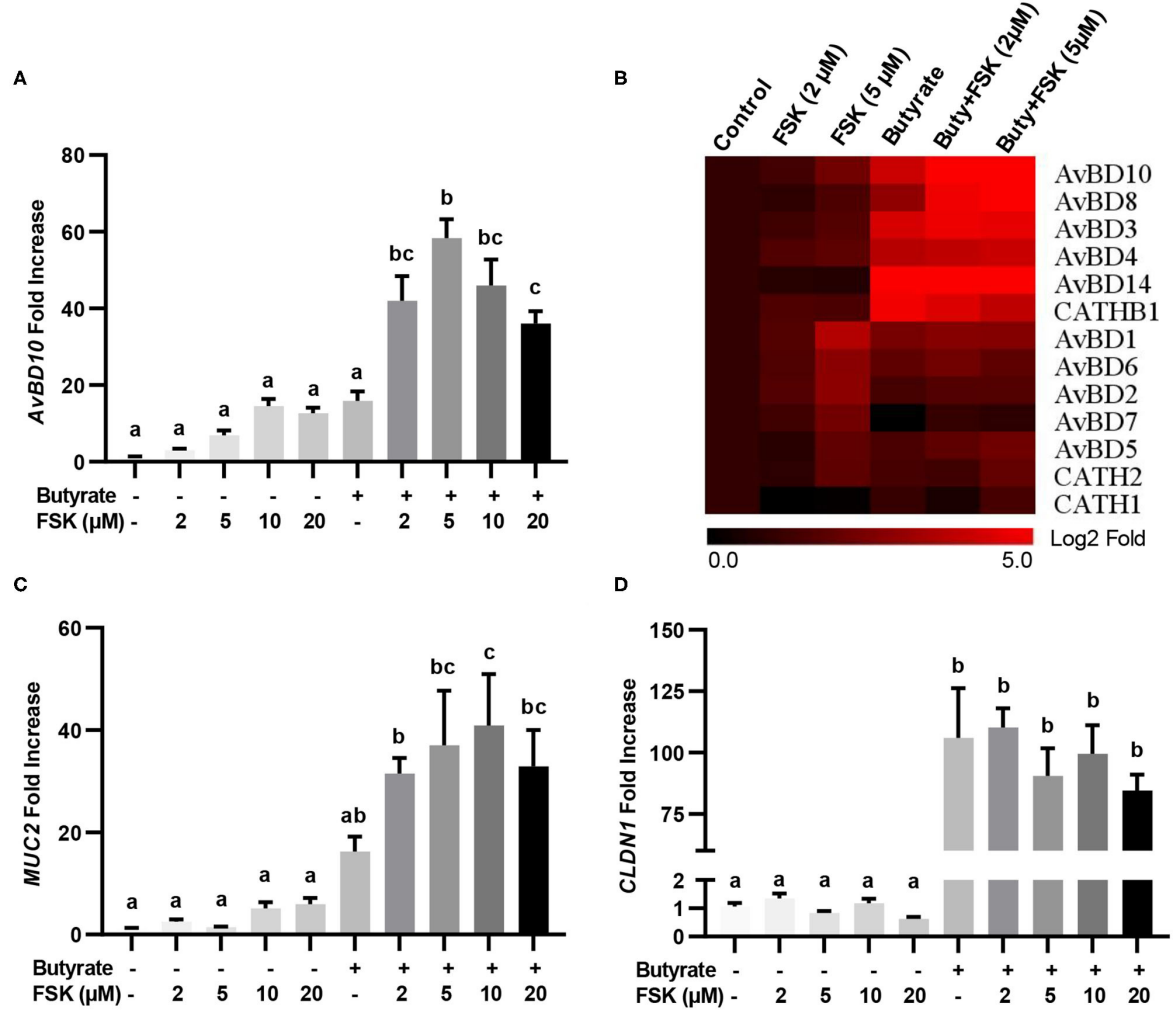
Synergistic induction of HDP and barrier function genes in macrophages by butyrate and FSK. Chicken HD11 cells were stimulated in duplicate with 2 mM butyrate and different concentrations of FSK individually or in combination for 24 h, followed by RT-qPCR analysis of *AvBD10* **(A)**, a panel of chicken HDP genes **(B)**, *MUC2* **(C)**, and *CLDN1* **(D)**. Fold changes in the heatmap were log_2_ transformed. Results are shown as means ± SEM of three independent experiments. Statistical significance (*P* < 0.05), denoted by non-common letters, was determined using one-way ANOVA and *post-hoc* Tukey's test.

The synergy between butyrate and FSK in regulating HDP and barrier function genes was further explored. Similar to *AvBD9* (18), *AvBD10* was also induced by butyrate and FSK in a synergistic manner. A peak 58-fold induction of *AvBD10* occurred in HD11 cells when 5 μM FSK was combined with 2 mM butyrate, while only 7- and 16-fold increases were observed when FSK and butyrate were applied separately (Figure 2A). Additionally, *AvBD3* and *AvBD8* were synergistically induced in response to butyrate and FSK (Figure 2B). Although all other chicken HDP genes were induced to different magnitudes in response to butyrate or FSK, no clear synergy was observed (Figure 2B). It is noteworthy that approximately a half number of HDP genes were more responsive to butyrate, while the other half were more readily induced by FSK (Figure 2B). Desirably, *MUC2* gene expression was synergistically enhanced by butyrate and FSK (Figure 2C), but no synergy was seen with *CLDN1* (Figure 2D).

**Figure 3:**
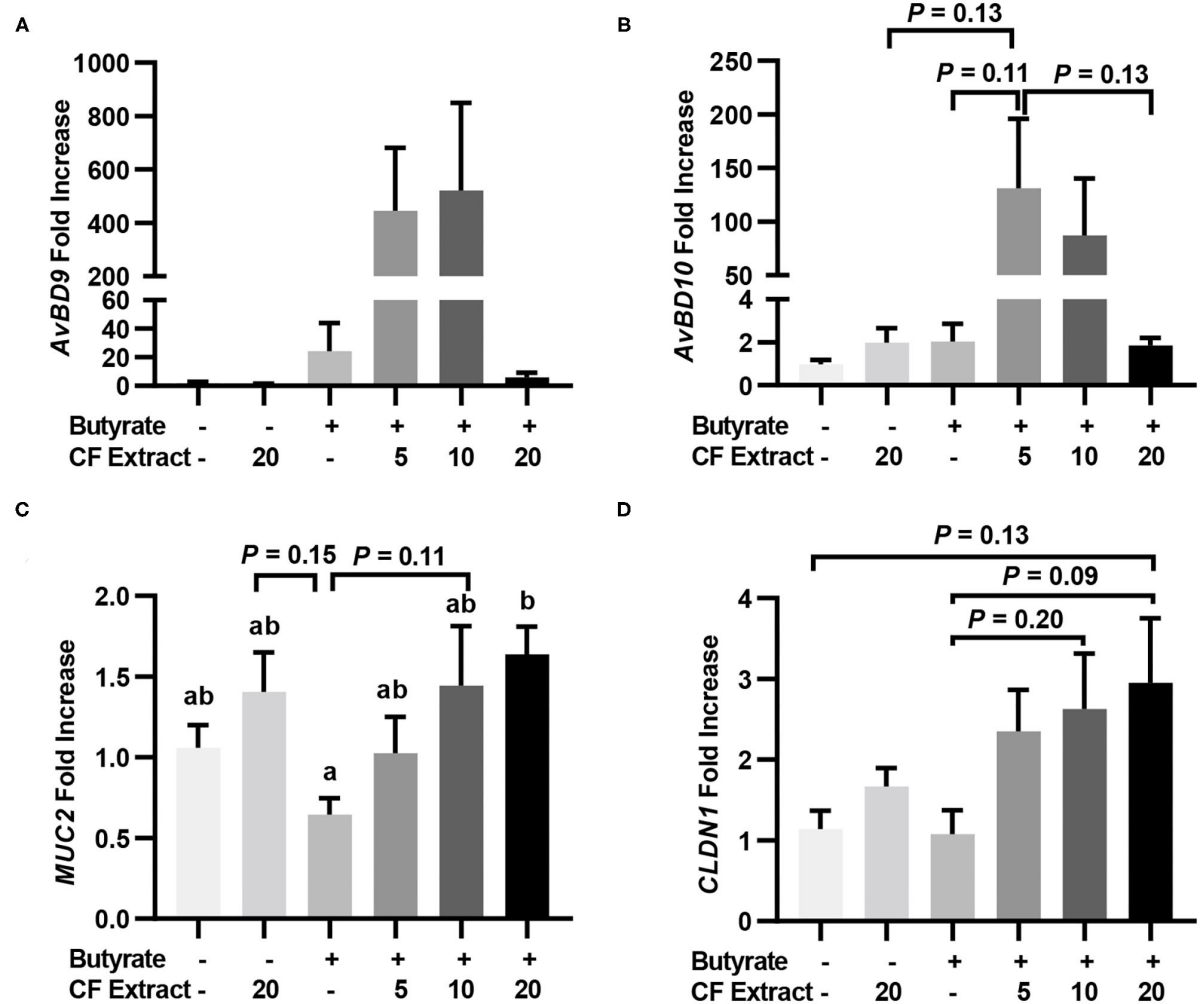
Induction of HDP and barrier function genes by butyrate and FSK in the jejunum of chickens. Five-day-old broiler chicks were fed a basal diet supplemented with or without 1 g/kg sodium butyrate or different concentrations of 10% FSK-containing *Coleus forskohlii* plant extract individually or in combination for 48 h. A segment of the jejunum was collected and subjected to RT-qPCR analysis of gene expressions of *AvBD9* **(A)**, *AvBD10* **(B)**, *MUC2* **(C)**, and *CLDN1* **(D)**. Results are shown as means ± SEM of eight chickens per group. Statistical significance (*P* < 0.05), denoted by non-common letters, was determined using one-way ANOVA and *post-hoc* Tukey's test. Treatments with *P* < 0.20 were also indicated to show a trend.

To verify whether HDP and barrier function gene induction would occur *in vivo* in response to butyrate and FSK, chickens were supplemented with 1 g/kg microencapsulated sodium butyrate or different concentrations of a 10% FSK-containing CF extract individually or in combination for 2 days before jejunal segments were harvested for RT-qPCR analysis of HDP and barrier function gene expression. CF extract at 20 mg/kg gave no or a minimum gene induction, while butyrate gave a modest 25- and 2-fold increase in *AvBD9* and *AvBD10*, respectively (Figures 3A,B). Importantly, a combination of butyrate and 5 mg/kg CF extract led to an average 445- and 131-fold increase in *AvBD9* and *AvBD10*, respectively (Figures 3A,B). *MUC2* and *CLDN1* expressions also tended to increase in the chicken jejunum in response to a combination of butyrate and FSK (Figures 3C,D).

### Butyrate and FSK Synergistically Alleviate NE in Chickens

**Figure 4:**
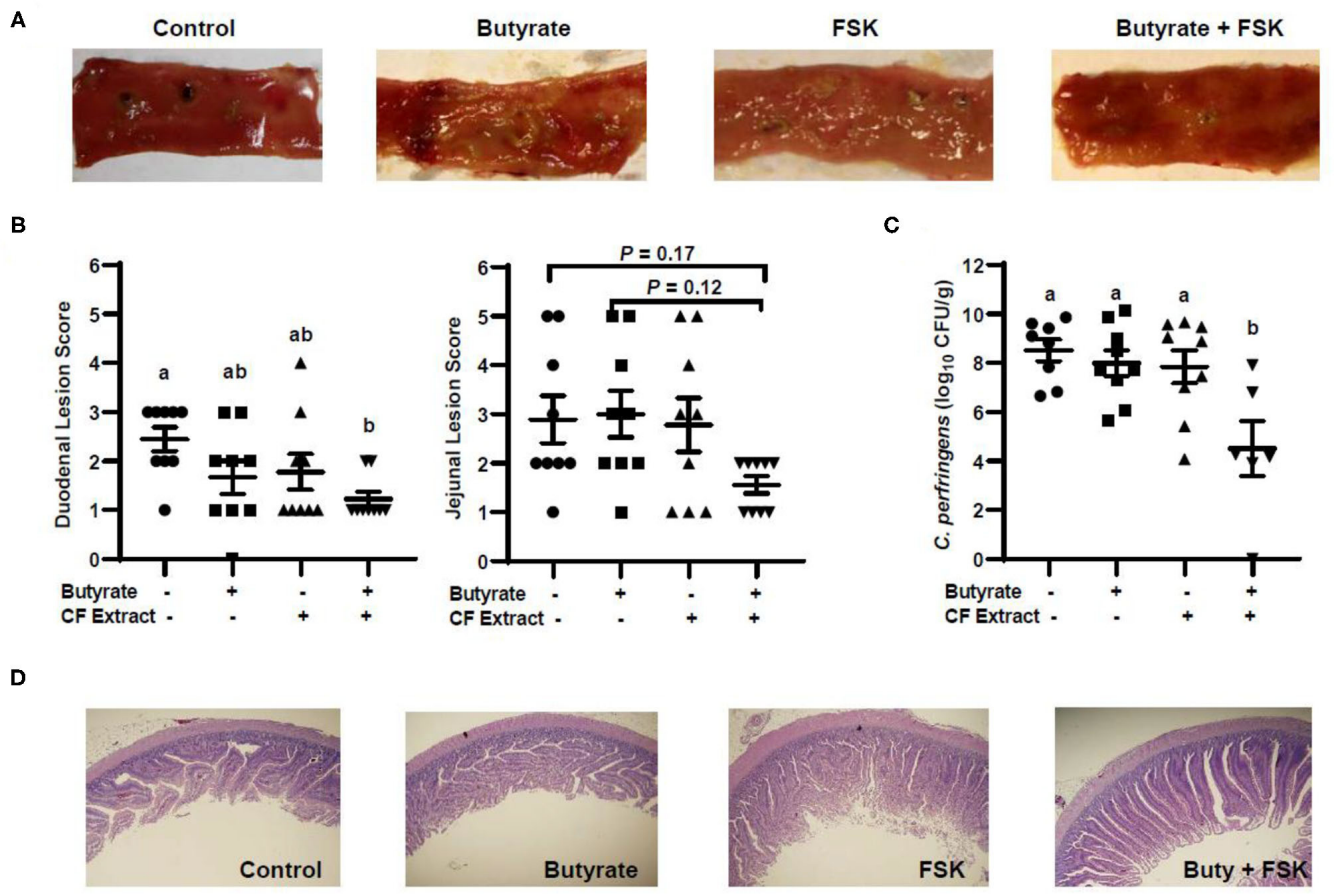
Alleviation of necrotic enteritis in chickens by butyrate and FSK. Chickens were fed a basal diet supplemented with or without 1 g/kg of sodium butyrate, 5 mg/kg of 10% FSK-containing *Coleus forskohlii* plant extract, or the combination for 3 days prior to induction of necrotic enteritis. After 4 days of infection, intestinal lesions, bacterial titer, and histology were examined in the duodenum and jejunum of each chicken. **(A)** Representative lesions in the jejunum of each group. **(B)** The lesions in the duodenum and jejunum were scored and compared among different groups (*n* = 9) 4 days post infection. **(C)** The titer of *Clostridium perfringens* in the jejunal contents of each animal. Statistical significance (*P* < 0.05), denoted by non-common letters, was determined using one-way ANOVA and *post-hoc* Tukey's test. Treatments with *P* < 0.20 were also indicated to show a trend. **(D)** Representative jejunal morphology of each group of chickens.

To evaluate the efficacy of butyrate and FSK in alleviating NE, chickens were supplemented with butyrate and FSK-containing CF extract individually or in combination for 4 days prior to daily challenges with *C. perfringens* to induce NE as described (28). As expected, the *C. perfringens* infection developed characteristic gross lesions in the small intestine (Figure 4A). Average lesion scores of 2.5 and 3 were observed in the duodenum and jejunum, respectively, if left unintervened (Figure 4B). Butyrate or FSK at the concentrations used tended to reduce the lesions in the duodenum, but not in the jejunum. Importantly, a combination of butyrate and FSK caused a significant decrease in the lesion score in the duodenum (*P* < 0.05) with a strong tendency also in the jejunum (*P* = 0.17) (Figure 4B). Similarly, butyrate and FSK alone failed to suppress *C. perfringens* colonization in the jejunum, and only the combination gave a significant, 4-log reduction in the bacterial titer (*P* < 0.05) (Figure 4C). Consistently, *C. perfringens* infection gave an obvious damage on the jejunal mucosa, and supplementation of butyrate or FSK alone showed no visible improvement, while only the combination was capable of restoring the epithelial integrity (Figure 4D). Overall, these results indicated a strong synergy between butyrate and FSK in ameliorating NE in broiler chickens.

### Butyrate and FSK Enhance Barrier Function Without Triggering Inflammation

**Figure 5:**
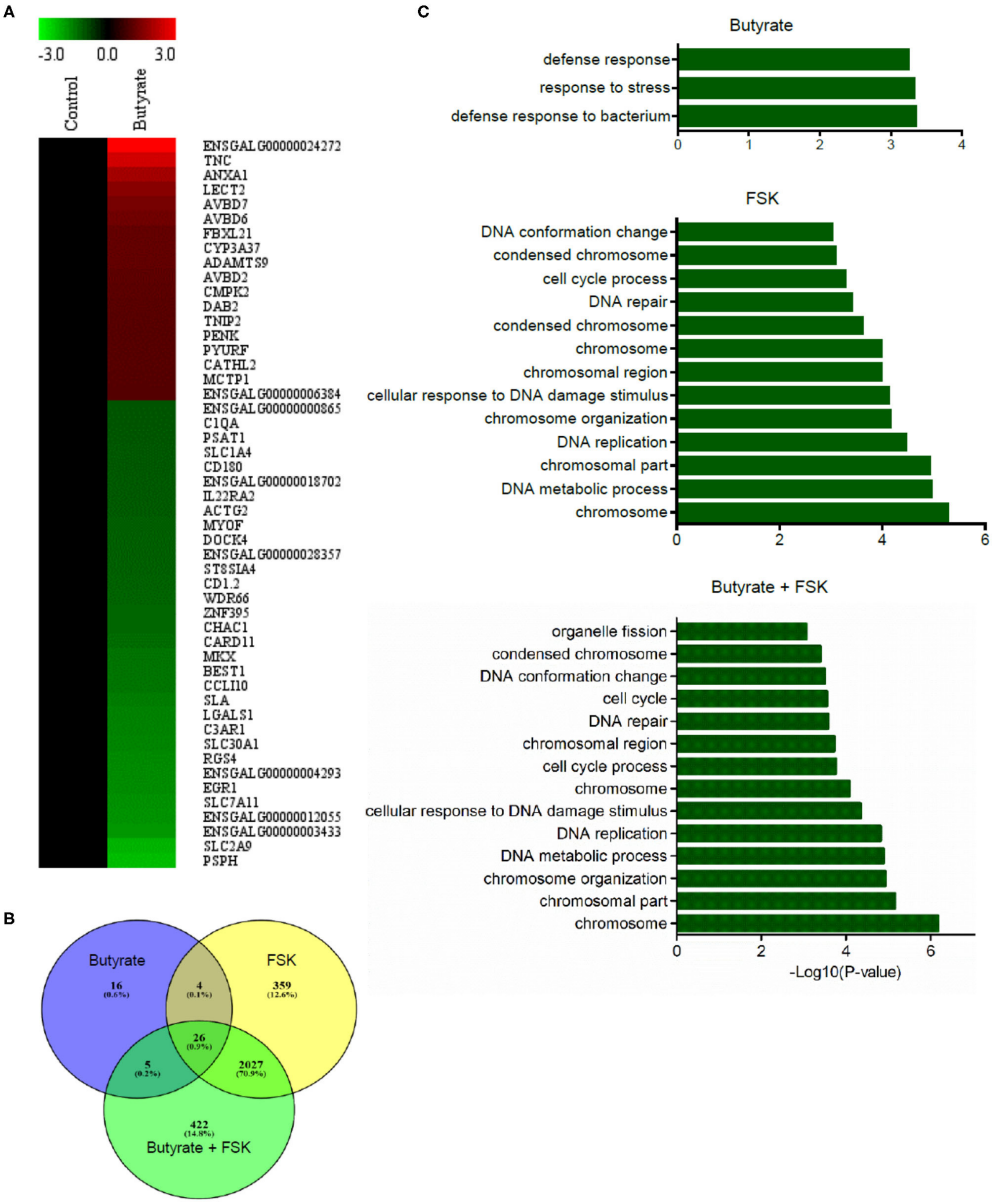
Transcriptome profiling of chicken macrophages in response to butyrate and FSK. Chicken HD11 cells were treated in duplicate with or without 2 mM butyrate, 5 μM FSK, or in combination for 24 h, followed by RNA isolation and sequencing. **(A)** Differential expression of genes in response to butyrate. Fold changes in the heatmap were log_2_ transformed. Red color denotes upregulation, while green color denotes downregulation. **(B)** Venn diagram showing the number of differentially expressed genes overlapping among the three treatments. Differentially expressed genes were selected with >2-fold difference (relative to the control) and FDR <0.05. **(C)** Differential enrichment of cellular processes to butyrate and FSK. Only the GO terms and KEGG pathways with *P* < 0.05 were considered significantly enriched.

To achieve a global understanding of the effect of butyrate and FSK on the transcriptome, RNA sequencing was performed with chicken HD11 cells following a 24-h treatment with or without butyrate, FSK or in combination. A total of 51 genes were found to be differentially regulated by butyrate, with 18 genes being upregulated and 33 downregulated (fold change > 2 and FDR < 0.05) (Figure 5A). Of note, four chicken HDP genes including *AvBD2, AvBD6, AvBD7*, and *CATH2*, were upregulated. FSK differentially regulated 2,416 genes, while the butyrate/FSK combination resulted in differential regulation of 2,480 genes (Figure 5B). Of all differentially regulated genes, a majority (2, 27) were regulated by both FSK and the butyrate/FSK combination, while 422 genes were uniquely regulated by the combination. Further evaluation of the cellular processes revealed that butyrate preferentially impacted defense and stress responses, while most processes affected by FSK and the butyrate/FSK combination were involved in DNA replication and repair and chromosomal organization (Figure 5C).

**Figure 6:**
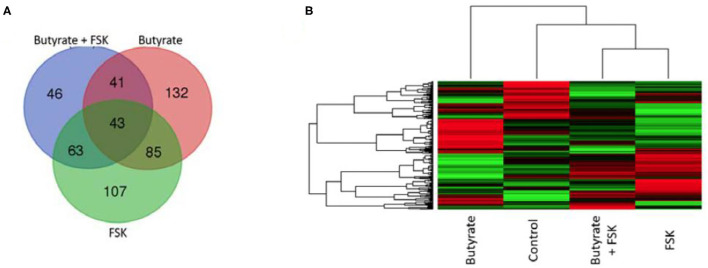
Kinome profiling of chicken macrophages in response to butyrate and FSK. Chicken HD11 cells were treated in duplicate with or without 2 mM butyrate, 5 μM FSK, or in combination for 4 h, followed by cell lysis and kinome peptide array analysis for differential phosphorylation status of kinases and phosphatases. **(A)** Venn diagram showing the number of differentially phosphorylated peptides overlapping among the three treatments. **(B)** Heatmap showing the profiles of differentially phosphorylated peptides in response to different treatments. Differentially regulated peptides were selected with *P* < 0.001 (relative to the control). Each horizontal line represents a peptide and its phosphorylation status, with red indicating phosphorylation and green indicating dephosphorylation.

To further understand a possible involvement of different signaling pathways in butyrate- and FSK-mediated response, HD11 cells were stimulated with butyrate and FSK individually or in combination for 4 h and then subjected to a chicken-specific kinome peptide array analysis as described (41). Among a total of 771 peptides analyzed, 301, 298, and 193 peptides were differentially phosphorylated by butyrate, FSK, and the combination, respectively (*P* < 0.001) (Figure 6A). Of those, 107 peptides were differentially phosphorylated only by the butyrate/FSK combination. A unique phosphorylation pattern was observed with each treatment, with FSK and the butyrate/FSK combination more resembling each other (Figure 6B). STRING analysis (42) further revealed that 116 and 100 pathways were significantly affected by butyrate and FSK, respectively, while 89 pathways were significantly altered by the combination (Supplementary Figure 1).

Of all genes and pathways regulated by a combination of butyrate and FSK, a special attention was given to those involved in barrier function and inflammation, given their significance in intestinal health. RNA sequencing revealed that several genes involved in barrier function such as *CLDN1* and *PAR6* were significantly induced by butyrate (FDR < 0.05), while FSK caused a significant reduction in *CLDN1* expression with a minimum impact on other tight junction genes (Supplementary Figure 2). A concomitant treatment with butyrate and FSK restored the beneficial effect of butyrate showing significant upregulation of several barrier function genes such as *CLDN1 CLDN5, CLDN10, PAR6*, and *MUC5B* (FDR < 0.05) (Supplementary Figure 2). Similarly, kinome peptide array showed that both butyrate and the butyrate/FSK combination induced a significant phosphorylation of multiple proteins involved in tight junction assembly such as Wiskott–Aldrich syndrome protein (WASP), RhoA, and Rho kinase (ROCK) (*P* < 0.001) (Supplementary Figure 3). These results suggested a beneficial role of butyrate and butyrate/FSK in enhancing barrier function. However, it is noted that junctional adhesion molecule 2 (*JAM2*) and *JAM3* were significantly downregulated by both butyrate and the butyrate/FSK combination (Supplementary Figure 2), and both proteins were dephosphorylated by butyrate, but restored in response to the butyrate/FSK combination (Supplementary Figure 3).

Butyrate and FSK are known to be antiinflammatory (12, 43). As expected, no pro- or antiinflammatory genes were significantly affected by butyrate, while interleukin-8 (*IL-8), IL-10*, and *IL-19* were induced by FSK, and *IL-8, IL-19*, and interferon-β (*IFN-*β) were augmented by the butyrate/FSK combination (data not shown). *IL-1*β and *IFN-*γ were not induced by either compound or their combination. Consistently, nuclear factor-κB (NF-κB) signaling pathway, a major pathway involved in inflammatory response (44), was not significantly affected by butyrate and FSK in either RNA sequencing or kinome peptide array analysis.

### ICER Is Induced by Butyrate and FSK and Suppresses HDP Gene Induction

**Figure 7:**
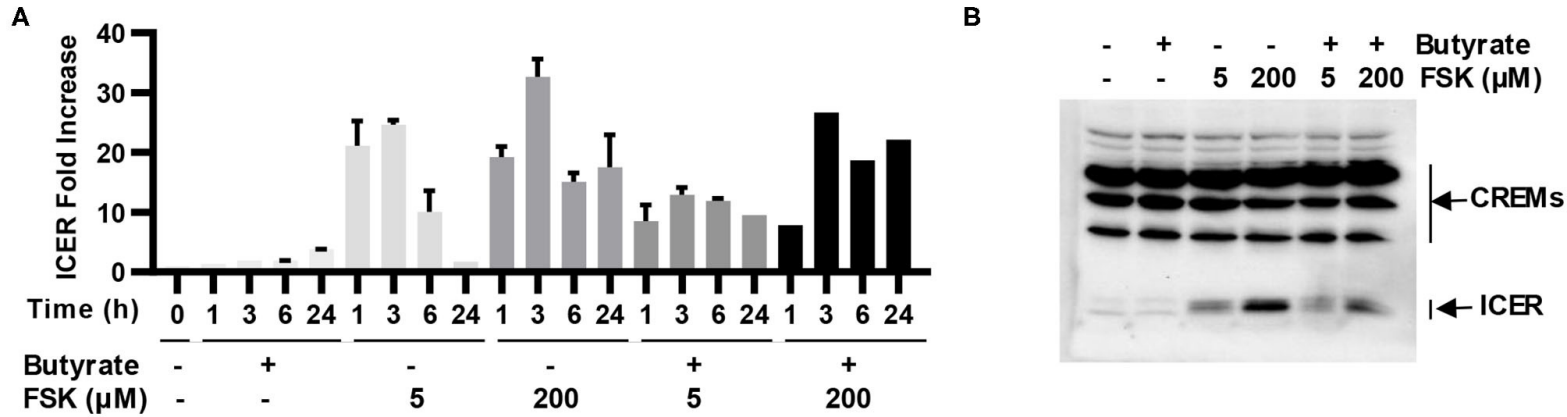
Induction of inducible cAMP early repressor (ICER) in chicken macrophages in response to butyrate and FSK. **(A)** Chicken HD11 cells were treated with 2 mM butyrate or two concentrations of FSK (5 and 200 μM) individually or in combination for indicated times, followed by RT-qPCR analysis of *ICER* expression. Results are shown as means ± SEM of three independent experiments. **(B)** HD11 cells were treated with 2 mM butyrate in the presence or absence of 5 or 200 μM FSK for 6 h, followed by Western blot analysis of ICER. Results are shown as a representative of two independent experiments.

ICER is a major suppressor of the cAMP-PKA-CREB pathway (19), which can otherwise be activated by FSK (15). To study whether ICER is involved in reduced synergy between butyrate and higher concentrations of FSK observed earlier in both cell culture (Figure 2A) and live animals (Figures 3A,B), HD11 cells were treated for various times with 2 mM butyrate and two different concentrations of FSK (5 and 200 μM) individually or in combination. It was apparent that both concentrations of FSK quickly induced *ICER* mRNA expression, with a peak 25-fold induction at 3 h. However, 200 μM FSK increased *ICER* transcription more strongly with a 32-fold upregulation at 3 h, and the induction was maintained at 18-fold even at 24 h, whereas the *ICER* expression was largely reduced to a basal level at 24 h in response to 5 μM FSK (Figure 7A). In contrast, *ICER* was gradually upregulated in response to butyrate in a time-dependent manner, with a minimum induction at 1–3 h and peaking at 24 h. *ICER* mRNA expression was maximum at 3 h, but remained substantially elevated at 24 in response to a combination of butyrate and FSK, with 200 μM FSK sustaining the *ICER* transcription by 21-fold at 24 h (Figure 7A). At the protein level, 200 μM FSK induced ICER synthesis at a much higher level than 5 μM FSK with or without butyrate (Figure 7B). These results suggested a reduced synergy between butyrate and higher concentrations of FSK observed earlier was likely due to a stronger induction of ICER.

## Discussion

In this study, we have demonstrated two natural products, butyrate and FSK, showing a synergistic activity in enhancing HDP genes as well as several major genes involved in barrier function without triggering inflammatory response. Furthermore, dietary supplementation of butyrate and FSK are synergistically protective against NE, a devastating enteric disease that costs $6 billion annually to the global poultry industry (23). The HDP-inducing, barrier-protective, and antiinflammatory properties of butyrate and FSK have been confirmed independently by qPCR, RNA sequencing, and kinome peptide array. For example, both qPCR and RNA sequencing have demonstrated an induction of multiple, but not all, HDP genes in response to butyrate and FSK. Differential response of HDP genes is likely reflected by close proximity of those genes. For example, *AvBD8, AvBD9*, and *AvBD10* are located in tandem on chicken chromosome 3 (45) and synergistically induced by butyrate and FSK. It is conceivable that these three HDP genes are likely originated from gene duplication and thus regulated similarly. It is interesting to note that approximately a half number of chicken HDP genes are highly responsive to butyrate, while the other half are responsive to FSK. Those FSK-responsive *AvBD* genes (*AvBD1/2/5/6/7*) are largely located continuously in one gene cluster on chromosome 3 that interrupts two clusters of butyrate-responsive genes (*AvBD8/19/10* and *AvBD3/4/14*) (45, 46). Another gene cluster (*AvBD11/12/13*) are barely detectable in HD11 cells and therefore, no results were shown for them. Among chicken cathelicidins, *CATHB1* is readily induced by butyrate, while *CATH1* and *CATH2* are more regulated by FSK. No synergy has been observed with any of those FSK-responsive HDP genes, while a half number of butyrate-responsive genes are induced by butyrate and FSK in a synergistic manner.

It is noted that nearly all chicken HDP genes are induced in HD11 cells in response to butyrate and FSK by RT-qPCR; however, RNA sequencing has only revealed significant upregulation of four (*AvBD2, AvBD6, AvBD7*, and *CATH2*). This apparent discrepancy is likely due to different sensitivities of the two technologies. RNA sequencing may not be sensitive enough to reliably detect those genes that are expressed in HD11 cells at extremely low levels. Furthermore, the threshold (FDR < 0.05 and fold change > 2) may be too stringent for a few HDP genes to be selected as differentially expressed genes. Nevertheless, both RT-qPCR and RNA sequencing highlight desirable preferential induction of multiple HDPs in response to butyrate and FSK.

Integrity of mucosal barrier function is maintained by the mucus layer and tight junctions (20). Butyrate is known to improve barrier integrity by inducing the expressions of *MUC2* and multiple proteins involved in tight junction assembly (12). In our study, qPCR confirmed induction of *MUC2, CLDN1*, and *CLDN5* by butyrate, and we showed for the first time that FSK is also capable of inducing MUC2, a predominant component of the mucus layer in the GI tract (21). Consistently, we demonstrated that butyrate and FSK cooperatively enhance *MUC2* expression both *in vitro* and *in vivo*. Although FSK suppresses *CLDN1* gene expression, a combination of butyrate and FSK reverses such an effect resulting in an upregulation of multiple claudins such as *CLDN1, CLDN5*, and *CLDN10* as revealed by RNA sequencing. Kinome peptide array further revealed butyrate- and FSK-mediated phosphorylation of many proteins that are involved in the assembly of tight junctions such as ROCK and WASP. ROCK is a serine-threonine kinase that regulates phosphorylation of myosin light chain and F-actin, thus affecting actin polymerization and cell contraction and tight junction assembly (47). Phosphorylation and activation of ROCK also activates p21-activated kinase, which in turn activates WASP (48), resulting in the nucleation and polymerization of actin filaments and reduced permeability of tight junctions (49).

Both butyrate and FKS have been shown to be primarily antiinflammatory (12, 43). Previous work in chicken HD11 cells has found butyrate to have no effect on *IL-1*β expression and a minimum effect on *IL-6, IL-8*, and *IL-12p40* (50, 51). Similarly, FSK showed no induction of *IL-1*β, while a combination of butyrate and FSK showed a minimum increase in *IL-1*β expression (data not shown). Consistently, RNA sequencing showed no significant induction of *IL-1*β or many other typical proinflammatory cytokine genes in response to butyrate and FSK. Furthermore, kinome peptide array indicated a minimum effect on the NF-κB pathway as well.

Butyrate is known to induce HDP expression mainly by functioning as HDACi to cause hyperacetylation of histones and hence chromatin relaxation (14). Consequently, a number of HDACi have been identified to enhance HDP expression (52–54). FSK is a natural agonist of adenylyl cyclase to activate the cAMP-PKA-CREB signaling pathway (15), which is involved in FSK-mediated HDP induction (17, 18). However, the mechanism underlying butyrate- and FSK-mediated synergy in HDP induction remains largely unknown, although both the mitogen-activated protein kinase and the cAMP-PKA pathways are involved (18).

Our preliminary studies indicated that neither FSK causes histone acetylation in HD11 cells, nor the butyrate/FSK combination leads to increased acetylation of histones relative to butyrate alone (data not shown), suggesting that histone hyperacetylation is not involved in HDP-inducing synergy between butyrate and FSK. However, biological processes such as DNA repair and chromosome organization are predominantly enriched in FSK- and butyrate/FSK-treated HD11 cells as revealed by RNA sequencing, suggesting that chromatin remodeling might be involved. Consistently, activation of the cAMP-PKA signaling was recently found to induce DNA damage and suppress DNA repair (55, 56), which subsequently help relieve DNA torsional stress and activate gene transcription at DNA damage sites, while global transcription is transiently silenced (57–59). Perhaps unsurprisingly, several small-molecule compounds that are inducers of DNA damage were recently found to upregulate HDP gene expression in a high throughput screening (53).

Furthermore, we have revealed a negative feedback mechanism involved in diminished HDP induction in response to higher concentrations of FSK with or without butyrate. ICER is transcriptionally induced by activation of the cAMP-PKA-CREB signaling pathway and serves as a major negative feedback mechanism to suppress gene transcription by competing with CREB for the same CRE region of the target gene promoter (19). We have found that ICER is quickly induced within 1 h, peaked at 3 h, and greatly subdued at 24 h in response to FSK or the FSK/butyrate combination, but higher concentrations of FSK keep ICER expression elevated even after 24 h, which is presumably responsible for reduced HDP gene expression.

## Conclusions

In summary, we have demonstrated a synergy in the induction of multiple, but not all, HDP genes between butyrate and FSK both *in vitro* and *in vivo*. Barrier function is also positively impacted. Desirably, butyrate and FSK enhance HDP expression and barrier integrity without causing inflammation. Consistently, butyrate and FSK are capable of ameliorating NE in a synergistic fashion. Therefore, butyrate and FSK, two natural products with HDP-inducing, barrier protective, and antiinflammatory activities, have the potential to be developed as novel antibiotic alternatives for disease control and prevention in poultry and possibly other animals.

## Data Availability Statement

The datasets presented in this study can be found in an online repository. The name of the repository and accession number can be found here: https://www.ncbi.nlm.nih.gov/sra/PRJNA763895.

## Ethics Statement

The animal study was reviewed and approved by the Institutional Animal Care and Use Committee at Oklahoma State University.

## Author Contributions

KR, QY, HL, LZ, BA, RA, and GZ conducted the experiments and processed all samples. KR, QY, BA, RA, and GZ performed data analysis. KR drafted the manuscript. GZ conceived the study and revised the manuscript. All authors contributed to the article and approved the submitted version.

## Funding

This research was funded by the USDA National Institute of Food and Agriculture (grant no. 2018-68003-27462 and 2020-67016-31619), Oklahoma Center for the Advancement of Science and Technology (grant no. AR19-027), the Ralph F. and Leila W. Boulware Endowment Fund, and Oklahoma Agricultural Experiment Station Project H-3112. The computing for this project was performed at the High Performance Computing Center at Oklahoma State University supported in part through the National Science Foundation grant OAC-1531128.

## Conflict of Interest

The authors declare that the research was conducted in the absence of any commercial or financial relationships that could be construed as a potential conflict of interest.

## Publisher's Note

All claims expressed in this article are solely those of the authors and do not necessarily represent those of their affiliated organizations, or those of the publisher, the editors and the reviewers. Any product that may be evaluated in this article, or claim that may be made by its manufacturer, is not guaranteed or endorsed by the publisher.

## Abbreviations

AvBD: avian β-defensin
cAMP: cyclic adenosine monophosphate
CF: *Coleus forskohlii*
CFU: colony-forming unit
CLDN1: claudin 1
CRE: cAMP responsive element
CREB: CRE-binding protein
CREM: CRE modulator
FDR: false discovery rate
FSK: forskolin
GI: gastrointestinal
GO: gene ontology
HDACi: histone deacetylase inhibitor
HDP: host defense peptide
ICER: inducible cAMP early repressor
IFN: interferon
IL: interleukin
KEGG: Kyoto Encyclopedia of Genes and Genomes
MUC2: mucin 2
NE: necrotic enteritis
NF-κB: nuclear factor-κB
PKA: protein kinase A
ROCK: Rho kinase
RT-qPCR: quantitative reverse transcription PCR
SCFA: short-chain fatty acid
SEM: standard error of the mean
WASP: Wiskott–Aldrich syndrome protein
ZO1: zonula occludens-1.

## References

[1] Watkins RR Bonomo RA. Overview: the ongoing threat of antimicrobial resistance. Infect Dis Clin North Am. (2020) 34:649–58. 10.1016/j.idc.2020.04.002 33011053

[2] Bergman P Raqib R Rekha RS Agerberth B Gudmundsson GH. Host directed therapy against infection by boosting innate immunity. Front Immunol. (2020) 11:1209. 10.3389/fimmu.2020.01209 32595649 PMC7304486

[3] Krug S Parveen S Bishai WR. Host-directed therapies: modulating inflammation to treat tuberculosis. Front Immunol. (2021) 12:660916. 10.3389/fimmu.2021.660916 33953722 PMC8089478

[4] Mookherjee N Anderson MA Haagsman HP Davidson DJ. Antimicrobial host defence peptides: functions and clinical potential. Nat Rev Drug Discov. (2020) 19:311–32. 10.1038/s41573-019-0058-8 32107480

[5] Ting DSJ Beuerman RW Dua HS Lakshminarayanan R Mohammed I. Strategies in translating the therapeutic potentials of host defense peptides. Front Immunol. (2020) 11:983. 10.3389/fimmu.2020.00983 32528474 PMC7256188

[6] Robinson K Ma X Liu Y Qiao S Hou Y Zhang G. Dietary modulation of endogenous host defense peptide synthesis as an alternative approach to in-feed antibiotics. Anim Nutr. (2018) 4:160–9. 10.1016/j.aninu.2018.01.003 30140755 PMC6104571

[7] Wu J Ma N Johnston LJ Ma X. Dietary nutrients mediate intestinal host defense peptide expression. Adv Nutr. (2020) 11:92–102. 10.1093/advances/nmz057 31204774 PMC7442325

[8] Xu D Lu W. Defensins: a double-edged sword in host immunity. Front Immunol. (2020) 11:764. 10.3389/fimmu.2020.00764 32457744 PMC7224315

[9] Scheenstra MR Van Harten RM Veldhuizen EJA Haagsman HP Coorens M. Cathelicidins modulate TLR-activation and inflammation. Front Immunol. (2020) 11:1137. 10.3389/fimmu.2020.01137 32582207 PMC7296178

[10] Cuperus T Coorens M Van Dijk A Haagsman HP. Avian host defense peptides. Dev Comp Immunol. (2013) 41:352–69. 10.1016/j.dci.2013.04.019 23644014

[11] Zhang G Sunkara LT. Avian antimicrobial host defense peptides: from biology to therapeutic applications. Pharmaceuticals. (2014) 7:220–47. 10.3390/ph7030220 24583933 PMC3978490

[12] Liu H Wang J He T Becker S Zhang G Li D. Butyrate: a double-edged sword for health? Adv Nutr. (2018) 9:21–9. 10.1093/advances/nmx009 29438462 PMC6333934

[13] Zhang L Liu C Jiang Q Yin Y. Butyrate in energy metabolism: there is still more to learn. Trends Endocrinol Metab. (2021) 32:159–69. 10.1016/j.tem.2020.12.003 33461886

[14] Kida Y Shimizu T Kuwano K. Sodium butyrate up-regulates cathelicidin gene expression via activator protein-1 and histone acetylation at the promoter region in a human lung epithelial cell line, EBC-1. Mol Immunol. (2006) 43:1972–81. 10.1016/j.molimm.2005.11.014 16423398

[15] Sapio L Gallo M Illiano M Chiosi E Naviglio D Spina A. The natural cAMP elevating compound forskolin in cancer therapy: is it time? J Cell Physiol. (2017) 232:922–7. 10.1002/jcp.25650 27739063

[16] Sands WA Palmer TM. Regulating gene transcription in response to cyclic AMP elevation. Cell Signal. (2008) 20:460–6. 10.1016/j.cellsig.2007.10.005 17993258

[17] Chakraborty K Maity PC Sil AK Takeda Y Das S. cAMP stringently regulates human cathelicidin antimicrobial peptide expression in the mucosal epithelial cells by activating cAMP-response element-binding protein, AP-1, and inducible cAMP early repressor. J Biol Chem. (2009) 284:21810–27. 10.1074/jbc.M109.001180 19531482 PMC2755907

[18] Sunkara LT Zeng X Curtis AR Zhang G. Cyclic AMP synergizes with butyrate in promoting beta-defensin 9 expression in chickens. Mol Immunol. (2014) 57:171–80. 10.1016/j.molimm.2013.09.003 24141182

[19] Rezen T Zmrzljak UP Bensa T Tomas TC Cirnski K Stojan J. Novel insights into biological roles of inducible cAMP early repressor ICER. Biochem Biophys Res Commun. (2020) 530:396–401. 10.1016/j.bbrc.2020.05.017 32534736

[20] Konig J Wells J Cani PD Garcia-Rodenas CL Macdonald T Mercenier A. Human intestinal barrier function in health and disease. Clin Transl Gastroenterol. (2016) 7:e196. 10.1038/ctg.2016.54 27763627 PMC5288588

[21] Paone P Cani PD. Mucus barrier, mucins and gut microbiota: the expected slimy partners? Gut. (2020) 69:2232–43. 10.1136/gutjnl-2020-322260 32917747 PMC7677487

[22] Zuo L Kuo WT Turner JR. Tight junctions as targets and effectors of mucosal immune homeostasis. Cell Mol Gastroenterol Hepatol. (2020) 10:327–40. 10.1016/j.jcmgh.2020.04.001 32304780 PMC7326733

[23] Caly DL D'inca R Auclair E Drider D. Alternatives to antibiotics to prevent necrotic enteritis in broiler chickens: a microbiologist's perspective. Front Microbiol. (2015) 6:1336. 10.3389/fmicb.2015.01336 26648920 PMC4664614

[24] Beug H Von Kirchbach A Doderlein G Conscience JF Graf T. Chicken hematopoietic cells transformed by seven strains of defective avian leukemia viruses display three distinct phenotypes of differentiation. Cell. (1979) 18:375–90. 10.1016/0092-8674(79)90057-6 227607

[25] Livak KJ Schmittgen TD. Analysis of relative gene expression data using real-time quantitative PCR and the 2[-Delta Delta C(T)] Method. Methods. (2001) 25:402–8. 10.1006/meth.2001.1262 11846609

[26] Jiang W Sunkara LT Zeng X Deng Z Myers SM Zhang G. Differential regulation of human cathelicidin LL-37 by free fatty acids and their analogs. Peptides. (2013) 50:129–38. 10.1016/j.peptides.2013.10.008 24140860

[27] Latorre JD Adhikari B Park SH Teague KD Graham LE Mahaffey BD. Evaluation of the epithelial barrier function and ileal microbiome in an established necrotic enteritis challenge model in broiler chickens. Front Vet Sci. (2018) 5:199. 10.3389/fvets.2018.00199 30186844 PMC6110846

[28] Cooper KK Songer JG. Virulence of *Clostridium perfringens* in an experimental model of poultry necrotic enteritis. Vet Microbiol. (2010) 142:323–8. 10.1016/j.vetmic.2009.09.065 19931323

[29] Shojadoost B Vince AR Prescott JF. The successful experimental induction of necrotic enteritis in chickens by *Clostridium perfringens*: a critical review. Vet Res. (2012) 43:74. 10.1186/1297-9716-43-74 23101966 PMC3546943

[30] Wang RF Cao WW Franklin W Campbell W Cerniglia CE. A 16S rDNA-based PCR method for rapid and specific detection of *Clostridium perfringens* in food. Mol Cell Probes. (1994) 8:131–7. 10.1006/mcpr.1994.1018 7935511

[31] Wu SB Rodgers N Choct M. Real-time PCR assay for *Clostridium perfringens* in broiler chickens in a challenge model of necrotic enteritis. Appl Environ Microbiol. (2011) 77:1135–9. 10.1128/AEM.01803-10 21148703 PMC3028694

[32] Martin M. Cutadapt removes adapter sequences from high-throughput sequencing reads. EMBnet J. (2011) 17:10–2. 10.14806/ej.17.1.200

[33] Schmieder R Edwards R. Quality control and preprocessing of metagenomic datasets. Bioinformatics. (2011) 27:863–4. 10.1093/bioinformatics/btr026 21278185 PMC3051327

[34] Kim D Pertea G Trapnell C Pimentel H Kelley R Salzberg SL. TopHat2: accurate alignment of transcriptomes in the presence of insertions, deletions and gene fusions. Genome Biol. (2013) 14:R36. 10.1186/gb-2013-14-4-r36 23618408 PMC4053844

[35] Li B Dewey CN. RSEM: accurate transcript quantification from RNA-Seq data with or without a reference genome. BMC Bioinformatics. (2011) 12:323. 10.1186/1471-2105-12-323 21816040 PMC3163565

[36] Robinson MD Mccarthy DJ Smyth GK. edgeR: a Bioconductor package for differential expression analysis of digital gene expression data. Bioinformatics. (2010) 26:139–40. 10.1093/bioinformatics/btp616 19910308 PMC2796818

[37] Howe E Holton K Nair S Schlauch D Sinha R Quackenbush J. MeV: multiexperiment viewer. In: Ochs M Casagrande J Davuluri R. Biomedical Informatics for Cancer Research. Boston, MA: Springer (2010). p. 267–77. 10.1007/978-1-4419-5714-6_15

[38] Bu D Luo H Huo P Wang Z Zhang S He Z. KOBAS-i: intelligent prioritization and exploratory visualization of biological functions for gene enrichment analysis. Nucleic Acids Res. (2021) 49:W317–25. 10.1093/nar/gkab447 34086934 PMC8265193

[39] Jalal S Arsenault R Potter AA Babiuk LA Griebel PJ Napper S. Genome to kinome: species-specific peptide arrays for kinome analysis. Sci Signal. (2009) 2:pl1. 10.1126/scisignal.254pl1 19155530

[40] Arsenault RJ Li Y Bell K Doig K Potter A Griebel PJ. *Mycobacterium avium* subsp. paratuberculosis inhibits gamma interferon-induced signaling in bovine monocytes: insights into the cellular mechanisms of Johne's disease. Infect Immun. (2012) 80:3039–48. 10.1128/IAI.00406-12 22689821 PMC3418731

[41] Li Y Arsenault RJ Trost B Slind J Griebel PJ Napper S. A systematic approach for analysis of peptide array kinome data. Sci Signal. (2012) 5:pl2. 10.1126/scisignal.2002429 22510468

[42] Szklarczyk D Gable AL Nastou KC Lyon D Kirsch R Pyysalo S. The STRING database in 2021: customizable protein-protein networks, and functional characterization of user-uploaded gene/measurement sets. Nucleic Acids Res. (2021) 49:D605–12. 10.1093/nar/gkaa1074 33237311 PMC7779004

[43] Chen Y Wen JG Feng JJ Wang YH Li TF Nurmi K. Forskolin attenuates the NLRP3 inflammasome activation and IL-1beta secretion in human macrophages. Pediatr Res. (2019) 86:692–8. 10.1038/s41390-019-0418-4 31086288

[44] Afonina IS Zhong Z Karin M Beyaert R. Limiting inflammation-the negative regulation of NF-kappaB and the NLRP3 inflammasome. Nat Immunol. (2017) 18:861–9. 10.1038/ni.3772 28722711

[45] Xiao Y Hughes AL Ando J Matsuda Y Cheng JF Skinner-Noble D. A genome-wide screen identifies a single beta-defensin gene cluster in the chicken: implications for the origin and evolution of mammalian defensins. BMC Genomics. (2004) 5:56. 10.1186/1471-2164-5-56 15310403 PMC515299

[46] Lynn DJ Higgs R Lloyd AT O'farrelly C Herve-Grepinet V Nys Y. Avian beta-defensin nomenclature: a community proposed update. Immunol Lett. (2007) 110:86–9. 10.1016/j.imlet.2007.03.007 17467809

[47] Jin Y Blikslager AT. The regulation of intestinal mucosal barrier by myosin light chain kinase/rho kinases. Int J Mol Sci. (2020) 21:3550. 10.3390/ijms21103550 32443411 PMC7278945

[48] Zhang W Bhetwal BP Gunst SJ. Rho kinase collaborates with p21-activated kinase to regulate actin polymerization and contraction in airway smooth muscle. J Physiol. (2018) 596:3617–35. 10.1113/JP275751 29746010 PMC6092288

[49] Alekhina O Burstein E Billadeau DD. Cellular functions of WASP family proteins at a glance. J Cell Sci. (2017) 130:2235–41. 10.1242/jcs.199570 28646090 PMC5536917

[50] Sunkara LT Achanta M Schreiber NB Bommineni YR Dai G Jiang W. Butyrate enhances disease resistance of chickens by inducing antimicrobial host defense peptide gene expression. PLoS ONE. (2011) 6:e27225. 10.1371/journal.pone.0027225 22073293 PMC3208584

[51] Sunkara LT Jiang W Zhang G. Modulation of antimicrobial host defense peptide gene expression by free fatty acids. PLoS ONE. (2012) 7:e49558. 10.1371/journal.pone.0049558 23166711 PMC3499459

[52] Deng Z Wang J Lyu W Wieneke X Matts R Ma X. Development of a cell-based high-throughput screening assay to identify porcine host defense peptide-inducing compounds. J Immunol Res. (2018) 2018:5492941. 10.1155/2018/5492941 30581875 PMC6276403

[53] Lyu W Deng Z Sunkara LT Becker S Robinson K Matts R. High throughput screening for natural host defense peptide-inducing compounds as novel alternatives to antibiotics. Front Cell Infect Microbiol. (2018) 8:191. 10.3389/fcimb.2018.00191 29942796 PMC6004375

[54] Rodriguez-Carlos A Jacobo-Delgado YM Santos-Mena AO Rivas-Santiago B. Modulation of cathelicidin and defensins by histone deacetylase inhibitors: a potential treatment for multi-drug resistant infectious diseases. Peptides. (2021) 140:170527. 10.1016/j.peptides.2021.170527 33744370

[55] Ben-Shlomo A Deng N Ding E Yamamoto M Mamelak A Chesnokova V. DNA damage and growth hormone hypersecretion in pituitary somatotroph adenomas. J Clin Invest. (2020) 130:5738–55. 10.1172/JCI138540 32673291 PMC7598090

[56] Noh SE Juhnn YS. Inhibition of non-homologous end joining of gamma ray-induced DNA double-strand breaks by cAMP signaling in lung cancer cells. Sci Rep. (2020) 10:14455. 10.1038/s41598-020-71522-9 32879366 PMC7468279

[57] Marnef A Cohen S Legube G. Transcription-coupled DNA double-strand break repair: active genes need special care. J Mol Biol. (2017) 429:1277–88. 10.1016/j.jmb.2017.03.024 28363678

[58] Puc J Aggarwal AK Rosenfeld MG. Physiological functions of programmed DNA breaks in signal-induced transcription. Nat Rev Mol Cell Biol. (2017) 18:471–6. 10.1038/nrm.2017.43 28537575 PMC5854152

[59] Long Q Liu Z Gullerova M. Sweet melody or jazz? Transcription around DNA double-strand breaks. Front Mol Biosci. (2021) 8:655786. 10.3389/fmolb.2021.655786 33959637 PMC8096065

