# Supplementary material for: Butyrate and Forskolin Augment Host Defense, Barrier Function, and Disease Resistance Without Eliciting Inflammation
Source: Front Nutr. 2021 Oct 27;8:778424. doi: 10.3389/fnut.2021.778424 (PMC8579826; doi:10.3389/fnut.2021.778424)
Supplement: Supplementary file 1 [file Data_Sheet_1.docx]

***Supplementary Material***

**Supplementary Figure 1.** Differential enrichment of KEGG pathways. Chicken HD11 cells were treated with or without 2 mM butyrate, 5 μM FSK, or in combination for 4 h, followed by cell lysis and chicken-specific kinome peptide array analysis. Differentially regulated pathways were selected with FDR < 0.001 (relative to the control).

**Supplementary Figure 2.** Differential enrichment of the genes that are involved in the tight junction assembly. Chicken HD11 cells were treated with or without 2 mM butyrate, 5 μM forksolin (FSK), or in combination for 24 h, followed by RNA isolation and sequencing. Differentially expressed genes were highlighted in red and selected with > 2-fold difference (relative to the control) and FDR < 0.05. Significantly upregulated genes are indicated by up arrows, whereas significantly downregulated genes are by down arrows.

**Supplementary Figure 3.** Differential phosphorylation of the proteins that are involved in the tight junction assembly. Chicken HD11 cells were treated with or without 2 mM butyrate, 5 μM forksolin (FSK), or in combination for 4 h, followed by cell lysis and chicken-specific kinome peptide array analysis. Differentially regulated peptides were selected with *P* < 0.001 (relative to the control). Significantly phosphorylated proteins are indicated by red color, whereas significant dephosphorylated proteins are by green color. Purple color means a protein that was detected with both significantly phosphorylated and dephosphorylated peptides, and therefore, its overall phosphorylation status remains unknown.
